# Extremal values on Zagreb indices of trees with given distance *k*-domination number

**DOI:** 10.1186/s13660-017-1597-3

**Published:** 2018-01-10

**Authors:** Lidan Pei, Xiangfeng Pan

**Affiliations:** School of Mathematical Sciences, Anhui University, Hefei, Anhui 230601 China

**Keywords:** 05C35, 05C69, first Zagreb index, second Zagreb index, trees, distance *k*-domination number

## Abstract

Let $G=(V(G),E(G))$ be a graph. A set $D\subseteq V(G)$ is a distance *k*-dominating set of *G* if for every vertex $u\in V(G)\setminus D$, $d_{G}(u,v)\leq k$ for some vertex $v\in D$, where *k* is a positive integer. The distance *k*-domination number $\gamma_{k}(G)$ of *G* is the minimum cardinality among all distance *k*-dominating sets of *G*. The first Zagreb index of *G* is defined as $M_{1}=\sum_{u\in V(G)}d^{2}(u)$ and the second Zagreb index of *G* is $M_{2}=\sum_{uv\in E(G)}d(u)d(v)$. In this paper, we obtain the upper bounds for the Zagreb indices of *n*-vertex trees with given distance *k*-domination number and characterize the extremal trees, which generalize the results of Borovićanin and Furtula (Appl. Math. Comput. 276:208–218, 2016). What is worth mentioning, for an *n*-vertex tree *T*, is that a sharp upper bound on the distance *k*-domination number $\gamma _{k}(T)$ is determined.

## Introduction

Throughout this paper, all graphs considered are simple, undirected and connected. Let $G=(V,E)$ be a simple and connected graph, where $V=V(G)$ is the vertex set and $E=E(G)$ is the edge set of *G*. The *eccentricity* of *v* is defined as $\varepsilon_{G}(v)=\max\{ d_{G}(u,v)\mid u\in V(G)\}$. The *diameter* of *G* is $\operatorname{diam}(G)=\max\{ \varepsilon_{G}(v)\mid v\in V(G)\}$. A path *P* is called a *diameter path* of *G* if the length of *P* is $\operatorname{diam}(G)$. Denote by $N_{G}^{i}(v)$ the set of vertices with distance *i* from *v* in *G*, that is, $N_{G}^{i}(v)=\{u\in V(G)\mid d(u,v)=i\}$. In particular, $N_{G}^{0}(v)=\{v\}$ and $N_{G}^{1}(v)=N_{G}(v)$. A vertex $v\in V(G)$ is called a *private* *k-neighbor* of *u* with respect to *D* if $\bigcup_{i=0}^{k}N^{i}_{G}(v)\cap D=\{u\}$. That is, $d_{G}(v,u)\leq k$ and $d_{G}(v,x)\geq k+1$ for any vertex $x\in D\setminus\{u\}$. The *pendent* vertex is the vertex of degree 1.

A chemical molecule can be viewed as a graph. In a molecular graph, the vertices represent the atoms of the molecule and the edges are chemical bonds. A topological index of a molecular graph is a mathematical parameter which is used for studying various properties of this molecule. The distance-based topological indices, such as the Wiener index [2, 3] and the Balaban index [4], have been extensively researched for many decades. Meanwhile the spectrum-based indices developed rapidly, such as the Estrada index [5], the Kirchhoff index [6] and matching energy [7]. The eccentricity-based topological indices, such as the eccentric distance sum [8], the connective eccentricity index [9] and the adjacent eccentric distance sum [10], were proposed and studied recently. The degree-based topological indices, such as the Randić index [11–13], the general sum-connectivity index [14, 15], the Zagreb indices [16], the multiplicative Zagreb indices [17, 18] and the augmented Zagreb index [19], where the Zagreb indices include the *first Zagreb index* $M_{1}=\sum_{u\in V(G)}d^{2}(u)$ and the *second Zagreb index* $M_{2}=\sum_{uv\in E(G)}d(u)d(v)$, represent one kind of the most famous topological indices. In this paper, we continue the work on Zagreb indices. Further study about the Zagreb indices can be found in [20–25]. Many researchers are interested in establishing the bounds for the Zagreb indices of graphs and characterizing the extremal graphs [1, 26–40].

A set $D\subseteq V(G)$ is a *dominating set* of *G* if, for any vertex $u\in V(G)\setminus D$, $N_{G}(u)\cap D\ne\emptyset$. The *domination number* $\gamma(G)$ of *G* is the minimum cardinality of dominating sets of *G*. For $k\in N^{+}$, a set $D\subseteq V(G)$ is a *distance* *k-dominating set* of *G* if, for every vertex $u\in V(G)\setminus D$, $d_{G}(u,v)\leq k$ for some vertex $v\in D$. The *distance* *k-domination number* $\gamma_{k}(G)$ of *G* is the minimum cardinality among all distance *k*-dominating sets of *G* [41, 42]. Every vertex in a minimum distance *k*-dominating set has a private *k*-neighbor. The domination number is the special case of the distance *k*-domination number for $k=1$. Two famous books [43, 44] written by Haynes *et al.* show us a comprehensive study of domination. The topological indices of graphs with given domination number or domination variations have attracted much attention of researchers [1, 45–47].

Borovićanin [1] showed the sharp upper bounds on the Zagreb indices of *n*-vertex trees with domination number *γ* and characterized the extremal trees. Motivated by [1], we describe the upper bounds for the Zagreb indices of *n*-vertex trees with given distance *k*-domination number and find the extremal trees. Furthermore, a sharp upper bound, in terms of $n,k$ and Δ, on the distance *k*-domination number $\gamma_{k}(T)$ for an *n*-vertex tree *T* is obtained in this paper.

## Lemmas

In this section, we give some lemmas which are helpful to our results.

### Lemma 2.1

([24, 48])

*If* *T* *is an* *n*-*vertex tree*, *different from the star* $S_{n}$, *then* $M_{i}(T)< M_{i}(S_{n})$ *for* $i=1,2$.

In what follows, we present two graph transformations that increase the Zagreb indices.

### Transformation I

([49])

**Figure 1:**
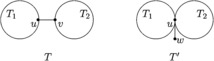
***T*** **and** $\pmb{T'}$ **in Transformation** **I** **.**

Let *T* be an *n*-vertex tree ($n>3$) and $e=uv\in E(T)$ be a nonpendent edge. Assume that $T-uv=T_{1}\cup T_{2}$ with vertex $u\in V(T_{1})$ and $v\in V(T_{2})$. Let $T'$ be the tree obtained by identifying the vertex *u* of $T_{1}$ with vertex *v* of $T_{2}$ and attaching a pendent vertex *w* to the *u* (=*v*) (see Figure 1). For the sake of convenience, we denote $T'=\tau(T,uv)$.

### Lemma 2.2

*Let* *T* *be a tree of order* *n* (≥3) *and* $T'=\tau(T,uv)$. *Then* $M_{i}(T')>M_{i}(T)$, $i=1,2$.

### Proof

It is obvious that $d_{T'}(u)=d_{T}(u)+d_{T}(v)-1$ and

$$\begin{aligned} M_{1}\bigl(T'\bigr)-M_{1}(T)={}& \bigl(d_{T}(u)+d_{T}(v)-1\bigr)^{2}+1-d_{T}^{2}(u)-d_{T}^{2}(v) \\ ={}&2\bigl(d_{T}(u)-1\bigr) \bigl(d_{T}(v)-1\bigr) \\ >{}&0.\end{aligned} $$

Let $x\in V(T)$ be a vertex different from *u* and *v*. Then

$$\begin{aligned} M_{2}\bigl(T'\bigr)-M_{2}(T)={}& \bigl(d_{T}(u)+d_{T}(v)-1\bigr) \biggl(\sum _{xu\in E(T_{1})}d_{T}(x)+\sum_{xv\in E(T_{2})}d_{T}(x)+1 \biggr) \\ &-d_{T}(u)\sum_{xu\in E(T_{1})}d_{T}(x)-d_{T}(v) \sum_{xv\in E(T_{2})}d_{T}(x) -d_{T}(u)d_{T}(v) \\ ={}&\bigl(d_{T}(v)-1\bigr)\sum_{xu\in E(T_{1})}d_{T}(x)+ \bigl(d_{T}(u)-1\bigr)\sum_{xv\in E(T_{2})}d_{T}(x) \\ &+d_{T}(u)+d_{T}(v)-1-d_{T}(u)d_{T}(v) \\ \geq{} &2\bigl(d_{T}(v)-1\bigr) \bigl(d_{T}(u)-1 \bigr)+d_{T}(u)+d_{T}(v)-1-d_{T}(u)d_{T}(v) \\ ={}&\bigl(d_{T}(v)-1\bigr) \bigl(d_{T}(u)-1\bigr) \\ >{}&0.\end{aligned} $$

This completes the proof. □

### Lemma 2.3

([50])

**Figure 2:**
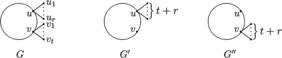
***G*** **,** $\pmb{G'}$ **and** $\pmb{G''}$ **in Lemma** **2.3** **.**

*Let* *u* *and* *v* *be two distinct vertices in* *G*. $u_{1},u_{2},\ldots,u_{r}$ *are the pendent vertices adjacent to* *u* *and* $v_{1}, v_{2},\ldots,v_{t}$ *are the pendent vertices adjacent to* *v*. *Define* $G'=G-\{vv_{1}, vv_{2},\ldots, vv_{t}\}+ \{uv_{1}, uv_{2},\ldots, uv_{t}\}$ *and* $G''=G-\{uu_{1}, uu_{2},\ldots, uu_{r}\}+\{ vu_{1}, vu_{2},\ldots, vu_{r}\}$, *as shown in Figure *2. *Then either* $M_{i}(G')>M_{i}(G)$ *or* $M_{i}(G'')>M_{i}(G)$, $i=1,2$.

### Lemma 2.4

([51])

*For a connected graph* *G* *of order* *n* *with* $n\geq k+1$, $\gamma_{k}(G)\leq\lfloor\frac {n}{k+1}\rfloor$.

Let *G* be a connected graph of order *n*. If $\gamma_{k}(G)\geq2$, then $n\geq k+1$. Otherwise, $\gamma_{k}(G)=1$, a contradiction. Hence, by Lemma 2.4, we have $\gamma_{k}(G)\leq\lfloor\frac{n}{k+1}\rfloor$ and $n\geq(k+1)\gamma_{k}$ for any connected graph *G* of order *n* if $\gamma _{k}(G)\geq2$.

### Lemma 2.5

*Let* *T* *be an* *n*-*vertex tree with distance* *k*-*domination number* $\gamma_{k}\geq2$. *Then* $\triangle \leq n-k\gamma_{k}$.

### Proof

Suppose that $\triangle\geq n-k\gamma_{k}+1$. Let $v\in V(T)$ be the vertex such that $d(v)=\triangle$ and $N(v)=\{v_{1},\ldots ,v_{\triangle}\}$. Denote by $T^{i}$ the component of $T-v$ containing the vertex $v_{i}$, $i=1,\ldots,\triangle$. Let *D* be a minimum distance *k*-dominating set of *T*,

$$S_{1}=\bigl\{ i\mid i\in\{1,2,\ldots,\triangle\}, 0\leq\varepsilon _{T^{i}}(v_{i})\leq k-1\bigr\} $$

and

$$S_{2}=\bigl\{ i\mid i\in\{1,2,\ldots,\triangle\}, \varepsilon _{T^{i}}(v_{i})\geq k\bigr\} . $$

Clearly, $|S_{2}|\geq1$. If not, $\{v\}$ is a distance *k*-dominating set of *T*, which contradicts $\gamma_{k}\geq2$. If $|S_{1}|=0$, then $\varepsilon_{T^{i}}(v_{i})\geq k$ for $i=1,\ldots ,\triangle$, so $|V(T^{i})\cap D|\geq1$. Therefore, $\gamma_{k}\geq\triangle\geq {n-k\gamma_{k}+1}$, which implies that $\gamma_{k}\geq\frac{n+1}{k+1}$. Since $\gamma_{k}\geq2$, $\gamma_{k}\leq\lfloor\frac{n}{k+1}\rfloor$ by Lemma 2.4, a contradiction. Thus, $|S_{1}|\geq1$. Let $i_{1}\in S_{1}$ and

$$\varepsilon_{T^{i_{1}}}(v_{i_{1}})=\max\bigl\{ \varepsilon_{T^{i}}(v_{i}) \mid i\in S_{1}\bigr\} =\lambda. $$

Then $0\leq\lambda\leq k-1$, so $|S_{2}|\leq\lfloor\frac{n-\triangle -1-\lambda}{k}\rfloor \leq\lfloor\frac{k\gamma_{k}-2}{k}\rfloor\leq\gamma_{k}-1$.

If $V(T^{i})\cap D=D_{1}\neq\emptyset$ for some $i\in S_{1}$, then $D-D_{1}+\{ v\}$ is a distance *k*-dominating set according to the definition of $S_{1}$. Thus, we assume that $V(T^{i})\cap D=\emptyset$ for each $i\in S_{1}$. Similarly, suppose that $D'\cap V(T^{i_{1}})=\emptyset$ where $D'$ is a minimum distance *k*-dominating set of the tree $T'=T-\bigcup_{i\in S_{1}\setminus\{i_{1}\}}V(T^{i})$.

We claim that $D'$ is a distance *k*-dominating set of *T*. Let $y\in V(T^{i_{1}})$ be the vertex such that $d(v_{i_{1}},y)=\lambda$ and $y'\in D'_{1}=\bigcup_{i=0}^{k}N_{T'}^{i}(y)\cap D'$. Then $y'\in V(T')\setminus V(T^{i_{1}})$ and $d(y,y')=d(y,v)+d(v,y')\leq k$, so, for $x\in\bigcup_{i\in S_{1}\setminus\{i_{1}\}}V(T^{i})$, we have $d(x,y')=d(x,v)+d(v,y')\leq d(y,v)+d(v,y')\leq k$. Hence, all the vertices in $\bigcup_{i\in S_{1}\setminus\{i_{1}\}}V(T^{i})$ can be dominated by $y'\in D'$. Therefore, $D'$ is a distance *k*-dominating set of *T*, so the claim is true.

In view of

$$k+1< (k+1)|S_{2}|+\lambda+2\leq\big|V\bigl(T'\bigr)\big|\leq n-|S_{1}|+1=n-\triangle+|S_{2}|+1, $$

one has

$$\begin{aligned} \gamma_{k}&\leq\big|D'\big| \\ &\leq\biggl\lfloor \frac{n-\triangle+|S_{2}|+1}{k+1}\biggr\rfloor \quad (\text{by Lemma 2.4}) \\ &\leq\biggl\lfloor \frac{(k+1)\gamma_{k}-1}{k+1}\biggr\rfloor \quad\bigl(\text{since }\triangle \geq n-k\gamma_{k}+1, |S_{2}|\leq\gamma_{k}-1\bigr) \\ &< \gamma_{k},\end{aligned} $$

a contradiction as desired. □

Determining the bound on the distance *k*-domination number of a connected graph is an attractive problem. In Lemma 2.5, an upper bound for the distance *k*-domination number of a tree is characterized. Namely, if *T* is an *n*-vertex tree with distance *k*-domination number $\gamma_{k}\geq2$, then $\gamma_{k}(T)\leq\frac{n-\Delta(T)}{k}$.

**Figure 3:**
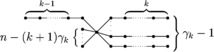
$\pmb{T_{n,k,\gamma_{k}}}$ **.**

Let $\mathscr{T}_{n,k,\gamma_{k}}$ be the set of all *n*-vertex trees with distance *k*-domination number $\gamma_{k}$ and $S_{n-k\gamma _{k}+1}$ be the star of order $n-k\gamma_{k}+1$ with pendent vertices $v_{1},v_{2},\ldots,v_{n-k\gamma_{k}}$. Denote by $T_{n,k,\gamma_{k}}$ the tree formed from $S_{n-k\gamma_{k}}$ by attaching a path $P_{k-1}$ to $v_{1}$ and attaching a path $P_{k}$ to $v_{i}$ for each $i\in\{2,\ldots,\gamma_{k}\}$, as shown in Figure 3. Then $T_{n,k,\gamma_{k}}\in\mathscr{T}_{n,k,\gamma_{k}}$. Even more noteworthy is the notion that $\gamma_{k}(T_{n,k,\gamma _{k}})=\gamma_{k}=\frac{n-\Delta(T_{n,k,\gamma_{k}})}{k}$. It implies that the upper bound on the distance *k*-domination number mentioned in the above paragraph is sharp.

The Zagreb indices of $T_{n,k,\gamma_{k}}$ are computed as

$$M_{1}(T_{n,k,\gamma_{k}})=(n-k\gamma_{k}) (n-k \gamma_{k}+1)+4(k\gamma_{k}-1) $$

and

$$M_{2}(T_{n,k,\gamma_{k}})= \textstyle\begin{cases} (n-k\gamma_{k})[n-(k-1)\gamma_{k}]+(4k-2)\gamma_{k}-4& \text{if }k\geq2,\\ 2(n-\gamma+1)(\gamma-1)+(n-\gamma)(n-2\gamma+1) &\text{if }k=1. \end{cases} $$

For $k=1$, the distance *k*-domination number $\gamma_{1}(G)$ is the domination number $\gamma(G)$. Furthermore, the upper bounds on the Zagreb indices of an *n*-vertex tree with domination number were studied in [1], so we only consider $k\geq2$ in the following.

### Lemma 2.6

([52])

*T* *be a tree on* $(k+1)n$ *vertices*. *Then* $\gamma_{k}(T)=n$ *if and only if at least one of the following conditions holds*:

1. *T* *is any tree on* $k+1$ *vertices*;
2. $T=R\circ k$ *for some tree* *R* *on* $n\geq1$ *vertices*, *where* $R\circ k$ *is the graph obtained by taking one copy of* *R* *and* $|V(R)|$ *copies of the path* $P_{k-1}$ *of length* $k-1$ *and then joining the* *ith vertex of* *R* *to exactly one end vertex in the* *ith copy of* $P_{k-1}$.

### Lemma 2.7

*Let* *T* *be an* *n*-*vertex tree with distance* *k*-*domination number* $\gamma_{k}(T)\geq3$. *If* $n=(k+1)\gamma _{k}$, *then*

$$M_{1}(T)\leq\gamma_{k}(\gamma_{k}+1)+4(k \gamma_{k}-1) $$

*and*

$$M_{2}(T)\leq2\gamma_{k}^{2}+(4k-2) \gamma_{k}-4, $$

*with equality if and only if* $T\cong T_{n,k,\gamma_{k}}$.

### Proof

When $n=(k+1)\gamma_{k}$, $T=R\circ k$ for some tree *R* on $\gamma_{k}$ vertices by Lemma 2.6. Assume that $V(R)=\{v_{1},\ldots,v_{\gamma_{k}}\}$. Then $d_{R}(v_{i})=d_{T}(v_{i})-1$. It is well known that $\sum_{i=1}^{n} d(u_{i})=2(n-1)$ for any *n*-vertex tree with vertex set $\{u_{1},\ldots ,u_{n}\}$. Hence, $\sum_{i=1}^{\gamma_{k}}d_{R}(v_{i})=2(\gamma_{k}-1)$. By the definition of the first Zagreb index, we have

$$\begin{aligned} M_{1}(T) =&\sum_{i=1}^{\gamma_{k}}d_{T}^{2}(v_{i})+ \sum_{x\in V(T)\setminus V(R)}d_{T}^{2}(x) \\ =&\sum_{i=1}^{\gamma_{k}}\bigl(d_{T}(v_{i})-1 \bigr)^{2}+\sum_{x\in V(T)\setminus V(R)}d_{T}^{2}(x)+2 \sum_{i=1}^{\gamma_{k}}\bigl(d_{T}(v_{i})-1 \bigr)+\gamma_{k} \\ =&M_{1}(R)+4(k-1)\gamma_{k}+\gamma_{k}+2\sum _{i=1}^{\gamma_{k}}d_{R}(v_{i})+ \gamma _{k} \\ \leq&M_{1}(S_{\gamma_{k}})+4(k-1)\gamma_{k}+2 \gamma_{k}+4(\gamma_{k}-1) \\ =&\gamma_{k}(\gamma_{k}+1)+4(k\gamma_{k}-1). \end{aligned}$$

The equality holds if and only if $R\cong S_{\gamma_{k}}$, that is, $T\cong T_{n,k,\gamma_{k}}$. We have

$$\begin{aligned} M_{2}(T)={}&\sum_{xy\in E(R)}d_{T}(x)d_{T}(y)+ \sum_{xy\in E(T)\setminus E(R)}d_{T}(x)d_{T}(y) \\ ={}&\sum_{xy\in E(R)}\bigl(d_{T}(x)-1\bigr) \bigl(d_{T}(y)-1\bigr)+\sum_{xy\in E(R)} \bigl(d_{T}(x)+d_{T}(y)-1\bigr) \\ &+\sum_{xy\in E(T)\setminus E(R)}d_{T}(x)d_{T}(y) \\ ={}&M_{2}(R)+\sum_{x\in V(R)}d_{T}(x) \bigl(d_{T}(x)-1\bigr)-(\gamma_{k}-1) \\ &+\sum_{x\in V(R)}2d_{T}(x)+4(k-2) \gamma_{k}+2\gamma_{k} \\ ={}&M_{2}(R)+\sum_{x\in V(R)} \bigl(d_{T}(x)-1\bigr)^{2} +3\sum _{x\in V(R)}\bigl(d_{T}(x)-1\bigr)+4k\gamma_{k}-5 \gamma_{k}-1 \\ ={}&M_{2}(R)+M_{1}(R)+6(\gamma_{k}-1)+4k \gamma_{k}-5\gamma_{k}+1 \\ \leq{}&M_{2}(S_{\gamma_{k}})+M_{1}(S_{\gamma_{k}})+4k \gamma_{k}+\gamma_{k}-5 \\ ={}&2\gamma_{k}^{2}+(4k-2)\gamma_{k}-4. \end{aligned} $$

The equality holds if and only if $R\cong S_{\gamma_{k}}$. As a consequence, $T\cong T_{n,k,\gamma_{k}}$. □

### Lemma 2.8

*Let* *G* *be a graph which has a maximum value of the Zagreb indices among all* *n*-*vertex connected graphs with distance* *k*-*domination number and* $S_{G}=\{v\in V(G)\mid d_{G}(v)=1,\gamma _{k}(G-v)=\gamma_{k}(G)\}$. *If* $S_{G}\neq\emptyset$, *then* $|N_{G}(S_{G})|=1$.

### Proof

Suppose that $|N_{G}(S_{G})|\geq2$ and *u* and *v* are two distinct vertices in $N_{G}(S_{G})$. $x_{1},x_{2},\ldots, x_{r}$ are the pendent vertices adjacent to *u* and $y_{1},y_{2},\ldots,y_{t}$ are the pendent vertices adjacent to *v*, where $r\geq1$ and $t\geq1$. Let *D* be a minimum distance *k*-dominating set of *G*. If $x_{i}\in D$ for some $i\in\{1,\ldots,r\}$, then $D-x_{i}+u$ is a distance *k*-dominating set of *T*. Hence, we assume that $x_{i}\notin D$, $i=1,\ldots,r$. Similarly, $y_{i}\notin D$ for $1\leq i\leq t$. Define $G_{1}=G-\{vy_{1}\}+\{uy_{1}\}$ and $G_{2}=G-\{ux_{1}\}+\{vx_{1}\}$. Then $\gamma_{k}(G_{1})=\gamma_{k}(G_{2})=\gamma_{k}(G)$. In addition, we have either $M_{i}(G_{1})>M_{i}(G)$ or $M_{i}(G_{2})>M_{i}(G)$, $i=1,2$, by a similar proof of Lemma 2.3 and thus omitted here (for reference, see the Appendix). It follows a contradiction, as desired. □

## Main results

In this section, we give upper bounds on the Zagreb indices of a tree with given order *n* and distance *k*-domination number $\gamma_{k}$. If $P=v_{0}v_{1}\cdots v_{d}$ is a diameter path of an *n*-vertex tree *T*, then denote by $T_{i}$ the component of $T-\{v_{i-1}v_{i},v_{i}v_{i+1}\} $ containing $v_{i}$, $i=1,2,\ldots,d-1$. By Lemma 2.1, we obtain Theorem 3.1 directly.

### Theorem 3.1

*Let* *T* *be an* *n*-*vertex tree and* $\gamma_{k}(T)=1$. *Then* $M_{1}(T)\leq n(n-1)$ *and* $M_{2}(T)\leq(n-1)^{2}$. *The equality holds if and only if* $T\cong S_{n}$.

Let $T^{i}_{n,k,2}$ be the tree obtained from the path $P_{2k+2}=v_{0}\cdots v_{2k+1}$ by joining $n-2(k+1)$ pendent vertices to $v_{i}$, where $i\in\{1,\ldots,2k\}$.

### Theorem 3.2

*If* *T* *is an* *n*-*vertex tree with distance* *k*-*domination number* $\gamma_{k}(T)=2$, *then*

$$M_{1}(T)\leq(n-2k) (n-2k+1)+4(2k-1), $$

*with equality if and only if* $T\cong T^{i}_{n,k,2}$, *where* $i\in\{1,\ldots,k\}$. *Also*,

$$M_{2}(T)\leq(n-2k) (n-2k+2)+8k-8, $$

*with equality if and only if* $T\cong T^{i}_{n,k,2}$, *where* $i\in\{2,\ldots,k\}$.

### Proof

Assume that $T\in\mathscr{T}_{n,k,2}$ is the tree that maximizes the Zagreb indices and $P=v_{0}v_{1}\cdots v_{d}$ is a diameter path of *T*. If $d\leq2k$, then $\{v_{\lfloor\frac{d}{2}\rfloor}\}$ is a distance *k*-dominating set of *T*, a contradiction to $\gamma_{k}(T)=2$. If $d\geq2k+2$, define $T'=\tau(T,v_{i}v_{i+1})$, where $i\in\{1,\ldots ,d-2\}$. Then $T'\in\mathscr{T}_{n,k,2}$. By Lemma 2.2, we have $M_{i}(T')>M_{i}(T)$, $i=1,2$, a contradiction. Hence, $d=2k+1$.

If $T_{i}$ is not a star for some $i\in\{1,2,\ldots,d-1\}$, then there exists an *n*-vertex tree $T'$ in $\mathscr{T}_{n,k,2}$ such that $M_{i}(T')>M_{i}(T)$ for $i=1,2$ by Lemma 2.2, a contradiction. Besides, $T\cong T^{i}_{n,k,2}$ for some $i\in\{1,\ldots,d-1\}$ by Lemma 2.3.

Since $M_{1}(T^{i}_{n,k,2})=M_{1}(T^{j}_{n,k,2})$ for $1\leq i\neq j\leq d-1$ and $T^{i}_{n,k,2}\cong T^{d-i}_{n,k,2}$ for $k+1\leq i\leq d-1$, we get $T\cong T^{i}_{n,k,2}$, $i\in\{1,\ldots,k\}$. By direct computation, one has $M_{1}(T)=M_{1}(T^{i}_{n,k,2})= (n-2k)(n-2k+1)+4(2k-1)$, $i\in\{1,\ldots ,k\}$. In addition, $M_{2}(T^{1}_{n,k,2})=M_{2}(T^{d-1}_{n,k,2})< M_{2}(T^{2}_{n,k,2})=\cdots =M_{2}(T^{d-2}_{n,k,2})$ and $T^{i}_{n,k,2}\cong T^{d-i}_{n,k,2}$ for $i\in \{k+1,\ldots,d-2\}$. Hence, $T\cong T^{i}_{n,k,2}$, where $i\in\{2,\ldots,k\}$. Moreover, $M_{2}(T)=M_{2}(T^{i}_{n,k,2})=(n-2k)(n-2k+2)+8k-8$. This completes the proof. □

### Lemma 3.3

*Let tree* $T\in\mathscr{T}_{n,k,3}$. *Then*

$$M_{1}(T)\leq(n-3k) (n-3k+1)+4(3k-1) $$

*and*

$$M_{2}(T)\leq(n-3k) (n-3k+3)+12k-10, $$

*with equality if and only if* $T\cong T_{n,k,3}$.

### Proof

Assume that $T\in\mathscr{T}_{n,k,3}$. We complete the proof by induction on *n*. By Lemma 2.4, we have $n\geq (k+1)\gamma_{k}$. This lemma is true for $n=(k+1)\gamma_{k}$ by Lemma 2.7. Suppose that $n>3(k+1)$ and the statement holds for $n-1$ in the following.

Let *D* be a minimum distance *k*-dominating set of *T* and $P=v_{0}v_{1}\cdots v_{d}$ be a diameter path of *T*. Then $d\geq2k+2$. Otherwise, $\{v_{k},v_{k+1}\}$ is a distance *k*-dominating set, a contradiction. Note that $\bigcup_{i=0}^{k}N_{T}^{i}(v_{0})\cap D\neq\emptyset$ and $\bigcup_{i=0}^{k}N_{T}^{i}(v_{0})\subseteq(\bigcup_{i=0}^{k-1}V(T_{i})\cup\{v_{k}\})$. Hence, $(\bigcup_{i=0}^{k-1}V(T_{i})\cup\{v_{k}\})\cap D\neq\emptyset$. However, $\bigcup_{i=0}^{k}N_{T}^{i}(x)\subseteq\bigcup_{i=0}^{k}N_{T}^{i}(v_{k})$ for $x\in\bigcup_{i=0}^{k}V(T_{i})\setminus\{v_{k}\} $, so we assume that $v_{k}\in D$ and $(\bigcup_{i=0}^{k}V(T_{i})\setminus\{ v_{k}\})\cap D=\emptyset$. Similarly, $v_{d-k}\in D$ and $(\bigcup_{i=d-k}^{d}V(T_{i})\setminus\{v_{d-k}\})\cap D=\emptyset$. Suppose that $v_{0}=u_{1}$, $v_{d}=u_{2},\ldots, u_{m}$ are the pendent vertices of *T* and $S_{T}=\{u_{i}\mid1\leq i\leq m, \gamma_{k}(T-u_{i})=\gamma_{k}(T)\}$. We have the following claim.

### Claim 1

$S_{T}\neq\emptyset$.

### Proof

Assume that $S_{T}=\emptyset$. Namely, $\gamma_{k}(T-u_{i})=\gamma_{k}(T)-1$ for each $i\in\{1,\ldots,m\}$. If $D\setminus\{w_{i}\}$ is a minimum distance *k*-dominating set of the tree $T-u_{i}$, where $w_{i}\in D$, then $w_{i}\neq w_{j}$ for $1\leq i\neq j\leq m$. Otherwise, $\gamma_{k}(T-u_{i})=\gamma_{k}(T)$ or $\gamma _{k}(T-u_{j})=\gamma_{k}(T)$, a contradiction. It follows that $m\leq\gamma_{k}$.

If $d_{T}(v_{i})\geq3$ for some $i\in\{2,\ldots,k,d-k,\ldots,d-1\}$, then $V(T_{i})\cap\{u_{3},\ldots,u_{m}\}\neq\emptyset$. In view of $\{ v_{k},v_{d-k}\}\subseteq D$, we have $\gamma_{k}(T-x)=\gamma_{k}(T)$ for $x\in V(T_{i})\cap\{u_{3},\ldots,u_{m}\}$, a contradiction. Hence, $d_{T}(v_{i})=2$ for $i\in\{2,\ldots,k,d-k,\ldots,d-1\}$.

Since $\gamma_{k}(T-v_{0})=\gamma_{k}(T)-1$, $v_{1}$ must be dominated by the vertices in $D\setminus\{v_{k}\}$. Bearing in mind that $(\bigcup_{i=0}^{k}V(T_{i})\setminus\{v_{k}\})\cap D=\emptyset$, one has $v_{k+1}\in D$. The same applies to $v_{d-k-1}$. Hence, $\{ v_{k},v_{k+1},v_{d-k-1},v_{d-k}\}\subseteq D$. If $d>2k+2$, then the vertices $v_{k}$, $v_{k+1}$, $v_{d-k-1}$ and $v_{d-k}$ are different from each other, a contradiction to $\gamma_{k}(T)=3$. As a consequence, $d=2k+2$ and thus $D=\{v_{k},v_{k+1},v_{d-k}\}$.

If $d_{T}(v_{k+1})=2$, then $T\cong P_{2k+3}$ and $\{v_{k},v_{d-k}\}$ is a distance *k*-dominating set, a contradiction. It follows that $d_{T}(v_{k+1})\geq3$. Hence, $m\geq3=\gamma_{k}$. Recalling that $m\leq \gamma_{k}=3$, we have $m=3$, which implies that $T_{k+1}$ is a path with end vertices $v_{k+1}$ and $u_{3}$. If $d(v_{k+1},u_{3})>k$, then $u_{3}$ cannot be dominated by the vertices in *D*. If $d(v_{k+1},u_{3})< k$, then $D\setminus\{v_{k+1}\}$ is a distance *k*-dominating set, a contradiction. Therefore, $d(v_{k+1},u_{3})=k$. We conclude that $|V(T)|=3(k+1)$, which contradicts $n>3(k+1)$, so Claim 1 is true. □

Considering $S_{T}\neq\emptyset$ for $T\in\mathscr{T}_{n,k,3}$, the tree among $\mathscr{T}_{n,k,3}$ that maximizes the Zagreb indices must be in the set $\{T^{*}\in\mathscr{T}_{n,k,3}\mid |N_{T^{*}}(S_{T^{*}})|=1\}$ by Lemma 2.8. To determine the extremal trees among $\mathscr{T}_{n,k,3}$, we assume that $T\in\{T^{*}\in\mathscr {T}_{n,k,3}\mid|N_{T^{*}}(S_{T^{*}})|=1\}$ in what follows.

Let $u_{i}$ be a pendent vertex such that $\gamma_{k}(T-u_{i})=\gamma_{k}(T)$ and *s* be the unique vertex adjacent to $u_{i}$. By Lemma 2.5, $d_{T}(s)\leq\triangle\leq n-k\gamma_{k}$. Define $A=\{x\in V(T)\mid d_{T}(x)=1, xs\notin E(T)\}$ and $B=\{x\in V(T)\mid d_{T}(x)\geq2, xs\notin E(T)\}$. Then $\gamma_{k}(T-x)=\gamma _{k}(T)-1$ for all $x\in A$. As a consequence, $|A|\leq\gamma_{k}$ from the proof of Claim 1. By the induction hypothesis,

$$\begin{aligned} M_{1}(T) =& M_{1}(T-u_{i})+2d(s) \\ \leq&(n-1-k\gamma_{k}) (n-1-k\gamma_{k}+1)+4(k \gamma_{k}-1)+2(n-k\gamma _{k}) \\ =&(n-k\gamma_{k}) (n-k\gamma_{k}+1)+4(k \gamma_{k}-1). \end{aligned}$$

The equality holds if and only if $T-u_{i}\cong T_{n-1,k,\gamma_{k}}$ and $d_{T}(s)=\triangle=n-k\gamma_{k}$, *i.e.*, $T\cong T_{n,k,\gamma_{k}}$.

Note that $|A|+|B|=n-1-d_{T}(s)$ and $|A|\leq\gamma_{k}$. Therefore, $|B|=n-1-d_{T}(s)-|A|\geq n-1-d_{T}(s)-\gamma_{k}$ and

$$\sum_{xs\notin E(T)}d(x)\geq|A|+2|B|=\bigl(|A|+|B|\bigr)+|B|\geq 2 \bigl(n-1-d_{T}(s)\bigr)-\gamma_{k}. $$

By the above inequality and the definition of $M_{2}$, we have

1 $$\begin{aligned} M_{2}(T) =&M_{2}(T-u_{i})+\sum _{v\in V(T)}d_{T}(v)-\sum_{xs\notin E(T)}d_{T}(x)-1 \\ \leq&M_{2}(T-u_{i})+2(n-1)-2\bigl(n-1-d_{T}(s) \bigr)+\gamma_{k}-1 \end{aligned}$$

2 $$\begin{aligned} \leq&(n-1-k\gamma_{k})\bigl[n-1-(k-1)\gamma_{k} \bigr]+(4k-2)\gamma_{k}-4 \\ &{}+2(n-k\gamma_{k})+\gamma_{k}-1 \quad\bigl(\text{since } d_{T}(s)\leq\triangle\leq n-k\gamma_{k}\bigr) \\ =&(n-k\gamma_{k})\bigl[n-(k-1)\gamma_{k}\bigr]+(4k-2) \gamma_{k}-4. \end{aligned}$$

The equality (1) holds if and only if $|A|=\gamma_{k}$, $|B|=n-1-d_{T}(s)-\gamma_{k}$ and $d_{T}(x)=2$ for $x\in B$. The equality (2) holds if and only if $T-u_{i}\cong T_{n-1,k,\gamma_{k}}$ and $d_{T}(s)=\triangle=n-k\gamma_{k}$. Hence, $M_{2}(T)\leq(n-k\gamma _{k})[n-(k-1)\gamma_{k}]+(4k-2)\gamma_{k}-4$ with equality if and only if $T\cong T_{n,k,\gamma_{k}}$. □

### Theorem 3.4

*Let* *T* *be a tree of order* *n* *with distance* *k*-*domination number* $\gamma_{k}$ (≥3). *Then*

$$M_{1}(T)\leq(n-k\gamma_{k}) (n-k\gamma_{k}+1)+4(k \gamma_{k}-1) $$

*and*

$$M_{2}(T)\leq(n-k\gamma_{k})\bigl[n-(k-1)\gamma_{k} \bigr]+(4k-2)\gamma_{k}-4, $$

*with equality if and only if* $T\cong T_{n,k,\gamma_{k}}$.

### Proof

Let $T\in\mathscr{T}_{n,k,\gamma_{k}}$ and $P=v_{0}v_{1}\cdots v_{d}$ be a diameter path of *T*. Define $S_{T}=\{u\in V(T)\mid d_{T}(u)=1,\gamma_{k}(T-u)=\gamma_{k}(T)\}$. If $S_{T}=\emptyset$, then $\gamma_{k}(T-v_{i})=\gamma_{k}(T)-1$ for $i=0,d$. If $S_{T}\neq\emptyset$, then we suppose that $T\in\{T^{*}\in\mathscr {T}_{n,k,\gamma_{k}}\mid|N_{T^{*}}(S_{T^{*}})|=1\}$ by Lemma 2.8 for establishing the maximum Zagreb indices of trees among $\mathscr {T}_{n,k,\gamma_{k}}$. If $v_{d}\in S_{T}\neq\emptyset$, then $\gamma_{k}(T-v_{0})=\gamma_{k}(T)-1$, which implies that $\gamma _{k}(T-v_{0})=\gamma_{k}(T)-1$ or $\gamma_{k}(T-v_{d})=\gamma_{k}(T)-1$. Assume that $\gamma_{k}(T-v_{0})=\gamma_{k}(T)-1$. Then there is a minimum distance *k*-dominating set *D* of *T* such that $\{v_{k},v_{k+1},v_{d-k}\}\subseteq D$ from the proof of Lemma 3.3.

**Figure 4:**
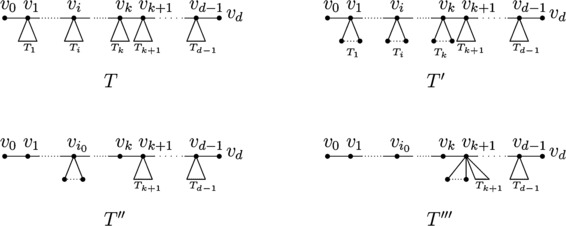
***T*** **,** $\pmb{T'}$ **,** $\pmb{T''}$ **and** $\pmb{T'''}$ **.**

Let $T'$ be the tree obtained from *T* by applying Transformation I on $T_{i}$ repeatedly for $i=1,\ldots,k$ such that $T'_{i}\cong S_{|V(T'_{i})|}$, where $T'_{i}$ is the component of $T'-\{ v_{i-1}v_{i},v_{i}v_{i+1}\}$ containing $v_{i}$, $i=1,\ldots,k$ (see Figure 4). Then $T'\in\mathscr{T}_{n,k,\gamma_{k}}$. By Lemma 2.2, we have $M_{i}(T)\leq M_{i}(T')$, $i=1,2$, with equality if and only if $T\cong T'$.

By Lemma 2.3, for some $i_{0},i_{1}\in\{1,\ldots,k\}$, define

$$\begin{aligned} T''={}&T'-\bigcup _{i\in\{1,\ldots,k\}\setminus\{i_{0}\}}\bigl\{ v_{i} x\mid x\in N_{T'}(v_{i}) \setminus\{v_{i-1},v_{i+1}\}\bigr\} \\ &+\bigcup_{i\in\{1,\ldots,k\}\setminus\{i_{0}\}}\bigl\{ v_{i_{0}}x\mid x\in N_{T'}(v_{i})\setminus\{v_{i-1},v_{i+1}\} \bigr\} \end{aligned} $$

and

$$\begin{aligned} \widetilde{T}''={}&T'-\bigcup _{i\in\{1,\ldots,k\}\setminus\{i_{1}\}}\bigl\{ v_{i} x\mid x\in N_{T'}(v_{i}) \setminus\{v_{i-1},v_{i+1}\}\bigr\} \\ & +\bigcup_{i\in\{1,\ldots,k\}\setminus\{i_{1}\}}\bigl\{ v_{i_{1}}x\mid x\in N_{T'}(v_{i})\setminus\{v_{i-1},v_{i+1}\} \bigr\} .\end{aligned} $$

Then one has $M_{1}(T')\leq M_{1}(T'')$ with equality if and only if $T'\cong T''$ and $M_{2}(T')\leq M_{2}(\widetilde{T}'')$ with equality if and only if $T'\cong\widetilde{T}''$.

Suppose that $|N_{T''}(v_{i_{0}})\setminus\{v_{i_{0}-1},v_{i_{0}+1}\} |=|N_{\widetilde{T}''}\setminus\{v_{i_{1}-1},v_{i_{1}+1}\}|=m$, $m\geq0$. Let

$$\begin{aligned} T'''={}&T''- \bigl\{ v_{i_{0}}x\mid x\in N_{T''}(v_{i_{0}})\setminus\{ v_{i_{0}-1},v_{i_{0}+1}\}\bigr\} \\ &+\bigl\{ v_{k+1}x\mid x\in N_{T''}(v_{i_{0}})\setminus \{v_{i_{0}-1},v_{i_{0}+1}\} \bigr\} \\ ={}&\widetilde{T}''-\bigl\{ v_{i_{1}}x\mid x\in N_{\widetilde {T}''}(v_{i_{1}})\setminus\{v_{i_{1}-1},v_{i_{1}+1}\} \bigr\} \\ &+\bigl\{ v_{k+1}x\mid x\in N_{\widetilde{T}''}(v_{i_{1}})\setminus \{ v_{i_{1}-1},v_{i_{1}+1}\}\bigr\} .\end{aligned} $$

Then *D* is a minimum distance *k*-dominating set of $T'''$ and $d_{T'''}(v_{i})=2$ for $i=1,\ldots,k$. Assume that $\mathit{PN}_{k,D}(x)$ is the set of all private *k*-neighbors of *x* with respect to *D* in $T'''$. It is clear that the vertices in $\bigcup_{i=0}^{k}N_{T'''}^{i}(v_{k})\setminus\{v_{0},\ldots,v_{k}\}$ can be dominated by $v_{k+1}\in D$. Thus, $D\setminus\{v_{k}\}$ is a distance *k*-dominating set of tree $T'''-\{v_{0},\ldots,v_{k}\}$. In addition, $\mathit{PN}_{k,D}(v_{k+1})\subseteq V(T''')\setminus\{v_{0},\ldots ,v_{k}\}$, which means that $D\setminus\{v_{k}\}$ is a minimum distance *k*-dominating set of $T'''-\{v_{0},\ldots,v_{k}\}$. So $\gamma_{k}(T'''-\{v_{0},\ldots,v_{k}\} )=\gamma_{k}-1$. Analogously, $\gamma_{k}(T'''-\{v_{0},\ldots,v_{k-1}\})=\gamma_{k}-1$.

By the definition of the first Zagreb index, we get

$$\begin{aligned} M_{1}\bigl(T''' \bigr)-M_{1}\bigl(T''\bigr)&=4+ \bigl(d_{T''}(v_{k+1})+m\bigr)^{2}-(2+m)^{2}-d_{T''}^{2}(v_{k+1}) \\ &=2m\bigl(d_{T''}(v_{k+1})-2\bigr) \\ &\geq0,\end{aligned} $$

so $M_{1}(T''')-M_{1}(T'')=0$ if and only if at least one of the following conditions holds:

1. $m=0$, which implies that $T''\cong T'''$;
2. $d_{T''}(v_{k+1})=2$.

If $i_{1}=1$, then

$$\begin{aligned} M_{2}\bigl(T''' \bigr)-M_{2}\bigl(\widetilde{T}''\bigr) ={}&6+ \bigl(d_{\widetilde{T}''}(v_{k+1})+m\bigr) \biggl(m+\sum _{x\in N_{\widetilde {T}''}(v_{k+1})}d_{\widetilde{T}''}(x)\biggr) \\ &-(m+2) (m+3)-d_{\widetilde{T}''}(v_{k+1})\sum _{x\in N_{\widetilde {T}''}(v_{k+1})}d_{\widetilde{T}''}(x) \\ ={}&m\biggl[d_{\widetilde{T}''}(v_{k+1})+\sum_{x\in N_{\widetilde {T}''}(v_{k+1})}d_{\widetilde{T}''}(x)-5 \biggr] \\ \geq{}&0 ,\end{aligned} $$

with equality if and only if $m=0$, that is, $\widetilde{T}''\cong T'''$. If $i_{1}\neq1$ and $i_{1}\neq k$, then

$$\begin{aligned} M_{2}\bigl(T''' \bigr)-M_{2}\bigl(\widetilde{T}''\bigr) ={}&8+ \bigl(d_{\widetilde{T}''}(v_{k+1})+m\bigr) \biggl(m+\sum _{x\in N_{\widetilde {T}''}(v_{k+1})}d_{\widetilde{T}''}(x)\biggr) \\ &-(m+2) (m+4)-d_{\widetilde{T}''}(v_{k+1})\sum _{x\in N_{\widetilde {T}''}(v_{k+1})}d_{\widetilde{T}''}(x) \\ ={}&m\biggl[d_{\widetilde{T}''}(v_{k+1})+\sum_{x\in N_{\widetilde {T}''}(v_{k+1})}d_{\widetilde{T}''}(x)-6 \biggr] \\ \geq{}&0 .\end{aligned} $$

Also, $M_{2}(T''')-M_{2}(\widetilde{T}'')=0$ if and only if at least one of the following conditions holds:

1. $m=0$, namely, $\widetilde{T}''\cong T'''$;
2. $d_{\widetilde{T}''}(v_{k})=d_{\widetilde {T}''}(v_{k+1})=d_{\widetilde{T}''}(v_{k+2})=2$.

If $i_{1}\neq1$ and $i_{1}=k$, then

$$\begin{aligned} M_{2}\bigl(T''' \bigr)-M_{2}\bigl(\widetilde{T}''\bigr) ={}&4+ \bigl(d_{\widetilde{T}''}(v_{k+1})+m\bigr) \biggl(m+2+\sum _{x\in N_{\widetilde {T}''}(v_{k+1})\setminus\{v_{k}\}}d_{\widetilde{T}''}(x)\biggr) \\ &-(m+2) (m+2)-d_{\widetilde{T}''}(v_{k+1}) \biggl(\sum _{x\in N_{\widetilde {T}''}(v_{k+1})\setminus\{v_{k}\}}d_{\widetilde{T}''}(x)+m+2\biggr) \\ ={}&m\biggl(\sum_{x\in N_{\widetilde{T}''}(v_{k+1})\setminus\{v_{k}\} }d_{\widetilde{T}''}(x)-2\biggr) \\ \geq{}&0 .\end{aligned} $$

As a result, $M_{2}(T''')-M_{2}(\widetilde{T}'')=0$ if and only if at least one of the following conditions holds:

1. $m=0$, which implies that $\widetilde{T}''\cong T'''$;
2. $d_{\widetilde{T}''}(v_{k+1})=d_{\widetilde{T}''}(v_{k+2})=2$.

In what follows, we prove $M_{1}(T''')\leq(n-k\gamma_{k})(n-k\gamma _{k}+1)+4(k\gamma_{k}-1)$ and $M_{2}(T''')\leq(n-k\gamma_{k})[n-(k-1)\gamma _{k}]+(4k-2)\gamma_{k}-4$ with equality if and only if $T'''\cong T_{n,k,\gamma_{k}}$ by induction on $\gamma_{k}$. The statement is true for $\gamma_{k}=3$ and $n\geq(k+1)\gamma_{k}$ by Lemma 3.3. Assume that $\gamma _{k}\geq4$, the statement holds for $\gamma_{k}-1$ and all the $n\geq (k+1)(\gamma_{k}-1)$.

In view of ${\gamma_{k}(T'''-\{v_{0},v_{1},\ldots,v_{k}\})}=\gamma _{k}-1$ and ${|V(T'''-\{v_{0},v_{1},\ldots,v_{k}\})|}=n-k-1\geq (k+1)(\gamma_{k}-1)$, by the induction hypothesis, we get

$$\begin{aligned} M_{1}\bigl(T'''\bigr)&= M_{1}\bigl(T'''- \{v_{0},v_{1},\ldots,v_{k}\} \bigr)+2d_{T'''}(v_{k+1})-1+ \sum_{i=0}^{k}d^{2}_{T'''}(v_{i}) \\ &\leq M_{1}(T_{n-k-1,k,\gamma_{k}-1})+2(n-k\gamma_{k})+4k \\ &=(n-k\gamma_{k}) (n-k\gamma_{k}+1)+4(k \gamma_{k}-1).\end{aligned} $$

The equality holds if and only if $T'''-\{v_{0},v_{1},\ldots,v_{k}\}\cong T_{n-k-1,k,\gamma_{k}-1}$ and $d_{T'''}(v_{k+1})=\triangle=n-k\gamma_{k}$. Recalling that $d_{T'''}(v_{i})=2$ for $i=1,\ldots,k$, we have $M_{1}(T''')=(n-k\gamma _{k})(n-k\gamma_{k}+1)+4(k\gamma_{k}-1)$ if and only if $T'''\cong T_{n,k,\gamma_{k}}$.

Thus, $M_{1}(T)\leq M_{1}(T')\leq M_{1}(T'')\leq M_{1}(T''')\leq(n-k\gamma _{k})(n-k\gamma_{k}+1)+4(k\gamma_{k}-1)$ and $M_{1}(T)=(n-k\gamma_{k})(n-k\gamma_{k}+1)+4(k\gamma_{k}-1)$ if and only if at least one of the following conditions holds:

1. $T\cong T'\cong T''\cong T'''\cong T_{n,k,\gamma_{k}}$;
2. $T\cong T'\cong T''$, where $d_{T''}(v_{k+1})=2$. Besides, $T'''\cong T_{n,k,\gamma_{k}}$.

However, the second condition is impossible. If $T'''\cong T_{n,k,\gamma _{k}}$, then $d_{T'''}(v_{k+1})=n-k\gamma_{k}$ and the number of the pendent vertices in $N_{T'''}(v_{k+1})$ is $n-(k+1)\gamma_{k}$. By the definition of $T'''$, we have

$$n-(k+1)\gamma_{k}\geq\big|N_{T''}(v_{i_{0}})\setminus \{v_{i_{0}-1},v_{i_{0}+1}\}\big|. $$

Hence,

$$\begin{aligned} d_{T''}(v_{k+1})&=d_{T'''}(v_{k+1})-|N_{T''}(v_{i_{0}}) \setminus\{ v_{i_{0}-1},v_{i_{0}+1}\}| \\ &\geq d_{T'''}(v_{k+1})-\bigl[n-(k+1)\gamma_{k}\bigr] \\ &=\gamma_{k}\geq3,\end{aligned} $$

a contradiction to $d_{T''}(v_{k+1})=2$. Therefore,

$$M_{1}(T)\leq(n-k\gamma_{k}) (n-k\gamma_{k}+1)+4(k \gamma_{k}-1) $$

with equality if and only if $T\cong T_{n,k,\gamma_{k}}$.

Note that ${\gamma_{k}(T'''-\{v_{0},\ldots,v_{k-1}\})}=\gamma _{k}-1$ and ${|V(T'''-\{v_{0},\ldots,v_{k-1}\})|}>(k+1)(\gamma _{k}-1)$. Then

$$\begin{aligned} M_{2}\bigl(T'''\bigr)&= M_{2}\bigl(T'''- \{v_{0},v_{1},\ldots,v_{k-1}\} \bigr)+d_{T'''}(v_{k+1})+4(k-1)+2 \\ &\leq M_{2}(T_{n-k,k,\gamma_{k}-1})+n-k\gamma_{k}+4(k-1)+2 \\ &=(n-k\gamma_{k})\bigl[n-(k-1)\gamma_{k}\bigr]+(4k-2) \gamma_{k}-4.\end{aligned} $$

The equality holds if and only if $T'''-\{v_{0},\ldots,v_{k-1}\}\cong T_{n-k,k,\gamma_{k}-1}$ and $d_{T'''}(v_{k+1})=\triangle=n-k\gamma _{k}$. In consideration of $d_{T'''}(v_{i})=2$ for $i=1,\ldots,k$, the equality holds if and only if $T'''\cong T_{n,k,\gamma_{k}}$.

Hence, if $i_{1}\neq1$, then $M_{2}(T)\leq M_{2}(T')\leq M_{2}(\widetilde {T}'')\leq M_{2}(T''')\leq(n-k\gamma_{k})[n-(k-1)\gamma_{k}]+(4k-2)\gamma _{k}-4$, with equality if and only if at least one of the following conditions holds:

1. $T\cong T'\cong\widetilde{T}''\cong T'''\cong T_{n,k,\gamma_{k}}$;
2. $T\cong T'\cong\widetilde{T}''$, where $d_{\widetilde {T}''}(v_{k})=d_{\widetilde{T}''}(v_{k+1})=d_{\widetilde {T}''}(v_{k+2})=2$ and $\widetilde{T}'''\cong T_{n,k,\gamma_{k}}$.

Analogous to the analysis of the first Zagreb index, the second condition above is impossible. Thus,

$$M_{2}(T)\leq(n-k\gamma_{k})\bigl[n-(k-1)\gamma_{k} \bigr]+(4k-2)\gamma_{k}-4 $$

and the equality holds if and only if $T\cong T_{n,k,\gamma_{k}}$.

Besides, if $i=1$, then $M_{2}(T)\leq(n-k\gamma_{k})[n-(k-1)\gamma _{k}]+(4k-2)\gamma_{k}-4$ with equality if and only if $T\cong T_{n,k,\gamma _{k}}$ immediately. This completes the proof. □

### Remark 3.5

Borovićanin and Furtula [1] proved

$$M_{1}(T)\leq(n-\gamma) (n-\gamma+1)+4(\gamma-1) $$

and

$$M_{2}(T)\leq 2(n-\gamma+1) (\gamma-1)+(n-\gamma) (n-2\gamma+1), $$

with equality if and only if $T\cong T_{n,\gamma}$, where $T_{n,\gamma}$ is the tree obtained from the star $K_{1,n-\gamma}$ by attaching a pendent edge to its $\gamma-1$ pendent vertices. In this paper, we determine the extremal values on the Zagreb indices of trees with distance *k*-domination number for $k\geq2$. Note that the domination number is the special case of the distance *k*-domination number for $k=1$ and $T_{n,k,\gamma_{k}}\cong T_{n,\gamma}$, $T_{n,k,2}^{i}\cong T_{n,\gamma}$, $i\in\{1,\ldots,k\}$, when $k=1$. Let *T* be an *n*-vertex tree with distance *k*-domination number $\gamma_{k}$. Then, by using Theorems 3.1, 3.2 and 3.4 and the results in [1], we have

$$M_{1}(T)\leq \textstyle\begin{cases}n(n-1) & \text{if }\gamma _{k}=1,\\ (n-k\gamma_{k})(n-k\gamma_{k}+1)+4(k\gamma_{k}-1) &\text{if }\gamma_{k}\geq2, \end{cases} $$

with equality if and only if $T\cong S_{n}$ when $\gamma_{k}=1$, $T\cong T_{n,k,2}^{i}$, $i\in\{1,\ldots,k\}$, when $\gamma_{k}=2$, or $T\cong T_{n,k,\gamma_{k}}$ when $\gamma_{k}\geq3$. Moreover,

$$M_{2}(T)\leq \textstyle\begin{cases}2(n-\gamma_{k}+1)(\gamma_{k}-1)+(n-\gamma_{k})(n-2\gamma _{k}+1) & \text{if }k=1,\\ (n-1)^{2} & \text{if }k\geq2, \gamma_{k}=1,\\ (n-k\gamma_{k})[n-(k-1)\gamma_{k}]+(4k-2)\gamma _{k}-4 & \text{if }k\geq2, \gamma_{k}\geq2, \end{cases} $$

with equality if and only if $T\cong S_{n}$ when $k\geq2$ and $\gamma _{k}=1$, $T\cong T_{n,k,2}^{i}$, $i\in\{2,\ldots,k\}$, when $k\geq2$ and $\gamma_{k}=2$, or $T\cong T_{n,k,\gamma_{k}}$ otherwise.

## Appendix

### Proof

Either $M_{i}(G_{1})>M_{i}(G)$ or $M_{i}(G_{2})>M_{i}(G)$, $i=1,2$, in Lemma 2.8, where $G_{1}=G-\{vy_{1}\}+\{uy_{1}\} $ and $G_{2}=G-\{ux_{1}\}+\{vx_{1}\}$, as shown in the following figure.  Let $G^{*}=G-\{x_{1},\ldots,x_{r},y_{1},\ldots,y_{t}\}$, $d_{G^{*}}(u)=a$ and $d_{G^{*}}(v)=b$. Then

$$\begin{aligned} M_{1}(G_{1})-M_{1}(G)&=(a+r+1)^{2}+(b+t-1)^{2}-(a+r)^{2}-(b+t)^{2} \\ &=2(a+r-b-t+1)\end{aligned} $$

and

$$\begin{aligned} M_{1}(G_{2})-M_{1}(G)&=(a+r-1)^{2}+(b+t+1)^{2}-(a+r)^{2}-(b+t)^{2} \\ &=2(b+t-a-r+1)\end{aligned} $$

by the definition of the first Zagreb index. Suppose that $M_{1}(G_{1})-M_{1}(G)\leq0$. Then $a+r\leq b+t-1$. It follows that $M_{1}(G_{2})-M_{1}(G)>0$.

If $u\notin N_{G}(v)$, then

$$\begin{aligned} M_{2}(G_{1})-M_{2}(G)={}&(a+r+1) \biggl(\sum _{x\in N_{G^{*}}(u)}d_{G}(x)+r+1 \biggr) \\ &+(b+t-1) \biggl(\sum_{x\in N_{G^{*}}(v)}d_{G}(x)+t-1 \biggr) \\ &-(a+r) \biggl(\sum_{x\in N_{G^{*}}(u)}d_{G}(x)+r \biggr)-(b+t) \biggl(\sum_{x\in N_{G^{*}}(v)}d_{G}(x)+t \biggr) \\ ={}&\sum_{x\in N_{G^{*}}(u)}d_{G}(x)-\sum _{x\in N_{G^{*}}(v)}d_{G}(x)+2r-2t+a-b+2\end{aligned} $$

and

$$\begin{aligned} M_{2}(G_{2})-M_{2}(G)={}&(a+r-1) \biggl(\sum _{x\in N_{G^{*}}(u)}d_{G}(x)+r-1 \biggr) \\ &+(b+t+1) \biggl(\sum_{x\in N_{G^{*}}(v)}d_{G}(x)+t+1 \biggr) \\ &-(a+r) \biggl(\sum_{x\in N_{G^{*}}(u)}d_{G}(x)+r \biggr)-(b+t) \biggl(\sum_{x\in N_{G^{*}}(v)}d_{G}(x)+t \biggr) \\ ={}&\sum_{x\in N_{G^{*}}(v)}d_{G}(x)-\sum _{x\in N_{G^{*}}(u)}d_{G}(x)+2t-2r+b-a+2.\end{aligned} $$

If $M_{2}(G_{1})-M_{2}(G)\leq0$, then $M_{2}(G_{2})-M_{2}(G)>0$.

If $u\in N_{G}(v)$, then

$$\begin{gathered} M_{2}(G_{1})-M_{2}(G) \\ \quad=(a+r+1) \biggl(\sum_{x\in N_{G^{*}}(u)\setminus\{v\}}d_{G}(x)+r+1 \biggr)+(b+t-1) \biggl(\sum_{x\in N_{G^{*}}(u)\setminus\{v\}}d_{G}(x)+t-1 \biggr) \\ \qquad{}+(a+r+1) (b+t-1)-(a+r) \biggl(\sum_{x\in N_{G^{*}}(u)\setminus\{v\} }d_{G}(x)+r \biggr) \\ \qquad{}-(b+t) \biggl(\sum_{x\in N_{G^{*}}(u)\setminus\{v\} }d_{G}(x)+t \biggr)-(a+r) (b+t) \\ \quad=\sum_{x\in N_{G^{*}}(u)\setminus\{v\}}d_{G}(x)-\sum _{x\in N_{G^{*}}(v)\setminus\{u\}}d_{G}(x)+r-t+1\end{gathered} $$

and

$$\begin{gathered} M_{2}(G_{2})-M_{2}(G) \\ \quad=(a+r-1) \biggl(\sum_{x\in N_{G^{*}}(u)\setminus\{v\}}d_{G}(x)+r-1 \biggr)+(b+t+1) \biggl(\sum_{x\in N_{G^{*}}(u)\setminus\{v\}}d_{G}(x)+t+1 \biggr) \\ \qquad{}+(a+r-1) (b+t+1)-(a+r) \biggl(\sum_{x\in N_{G^{*}}(u)\setminus\{v\} }d_{G}(x)+r \biggr) \\ \qquad{}-(b+t) \biggl(\sum_{x\in N_{G^{*}}(u)\setminus\{v\}}d_{G}(x)+t \biggr)-(a+r) (b+t) \\ \quad=\sum_{x\in N_{G^{*}}(v)\setminus\{u\}}d_{G}(x)-\sum _{x\in N_{G^{*}}(u)\setminus\{v\}}d_{G}(x)+t-r+1.\end{gathered} $$

Assume that $M_{2}(G_{1})-M_{2}(G)\leq0$. Then $M_{2}(G_{2})-M_{2}(G)>0$. Therefore, either $M_{i}(G_{1})>M_{i}(G)$ or $M_{i}(G_{2})>M_{i}(G)$, $i=1,2$. □
